# Supplementary material for: Differences in homologous recombination and maintenance of heteropolyploidy between Haloferax volcanii and Haloferax mediterranei
Source: G3 (Bethesda). 2022 Dec 1;13(4):jkac306. doi: 10.1093/g3journal/jkac306 (PMC10085750; doi:10.1093/g3journal/jkac306)
Supplement: jkac306_Supplementary_Data [file jkac306_supplementary_data.zip › Supplemental_Material_Legends_G3-2022-403706.docx]

**Supplementary Figures Legends**

**Supplementary Figure 1** Two pairs of strains were generated, an *H. volcanii* pair and an *H. mediterranei* pair. The pairs were employed in mating assays (either *volcanii-volcanii* or *mediterranei*-*mediterranei* mating). The two strains were constructed such that the *trpA* or *hdrB* cassettes were inserted back into the same ectopic location, instead of the ASC-like gene HVO1585, which was previously demonstrated to be non-essential. This genetic background allowed for the selection of mated cells on growth medium lacking tryptophan and thymidine. The mated cells are thus forced to contain two different types of genomes and are essentially held under selection in a heteropolyploid state. A deletion in the *crtI* gene, resulted in a white phenotype in contrast with the other strain that remained naturally pigmented (red) in each pair. Upon plating on rich medium, cells are no longer forced to withhold both genomes and resolve to a ‘homopolyploid’ state can either show the ‘parental’ genome or a recombinant genome shown below.

**Supplementary Figure 2.** Similarity to parent strains among homopolyploid cells after mating and subsequent loss of heteropolyploidy. Representative PCR results. 5% of the colonies that were considered homopolyploid after streaking were also tested by PCR on locus 1585 of H. volcanii and 1643 of H. mediterranei. The results completely matched the observed phenotype.

**Supplementary File Legend**

"Supplementary File 1: Comparing RNA expression between *H. volcanii* and *H. mediterranei*. Tab 1, labelled "Data comparison", compares RNA expression levels for log and stationary phase cultures of *H. volcanii* and *H. mediterranei*. This is denoted by RPKM values (Reads Per Kb transcript per Million reads). Fold differences between both species are highlighted in brackets. Tabs 2 and 3 contain gene-level RPKM values for log and stationary phase *H. mediterranei*cultures, respectively*.*Tab 4 (labelled "Hvol data at log and stationary") contains similar gene level data for log and stationary phase *H. volcanii*cultures. Tab 5, labelled "Hvol gene names", provides additional information on gene names for *H. volcanii*."
