# Supplementary figures and images for: Differences in homologous recombination and maintenance of heteropolyploidy between Haloferax volcanii and Haloferax mediterranei
Source: G3 (Bethesda). 2022 Dec 1;13(4):jkac306. doi: 10.1093/g3journal/jkac306 (PMC10085750; doi:10.1093/g3journal/jkac306)

Supplementary Figure 2

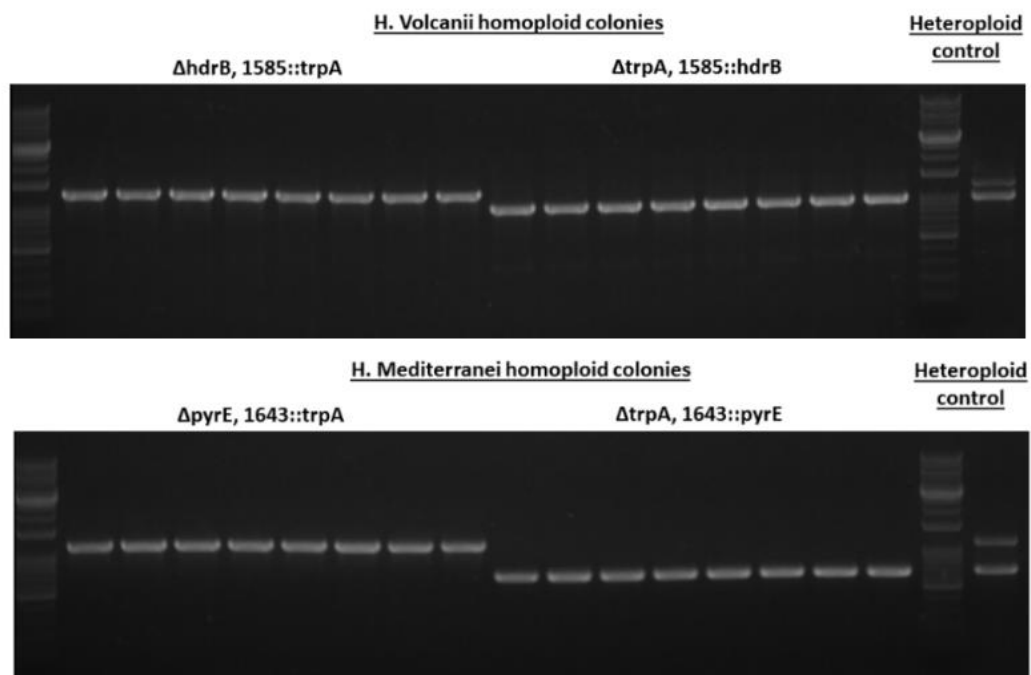

Supplement: jkac306_Supplementary_Data [file jkac306_supplementary_data.zip › Supplementary_Figure_2_G3-2022-403706.pdf]

Supplementary Figure 1

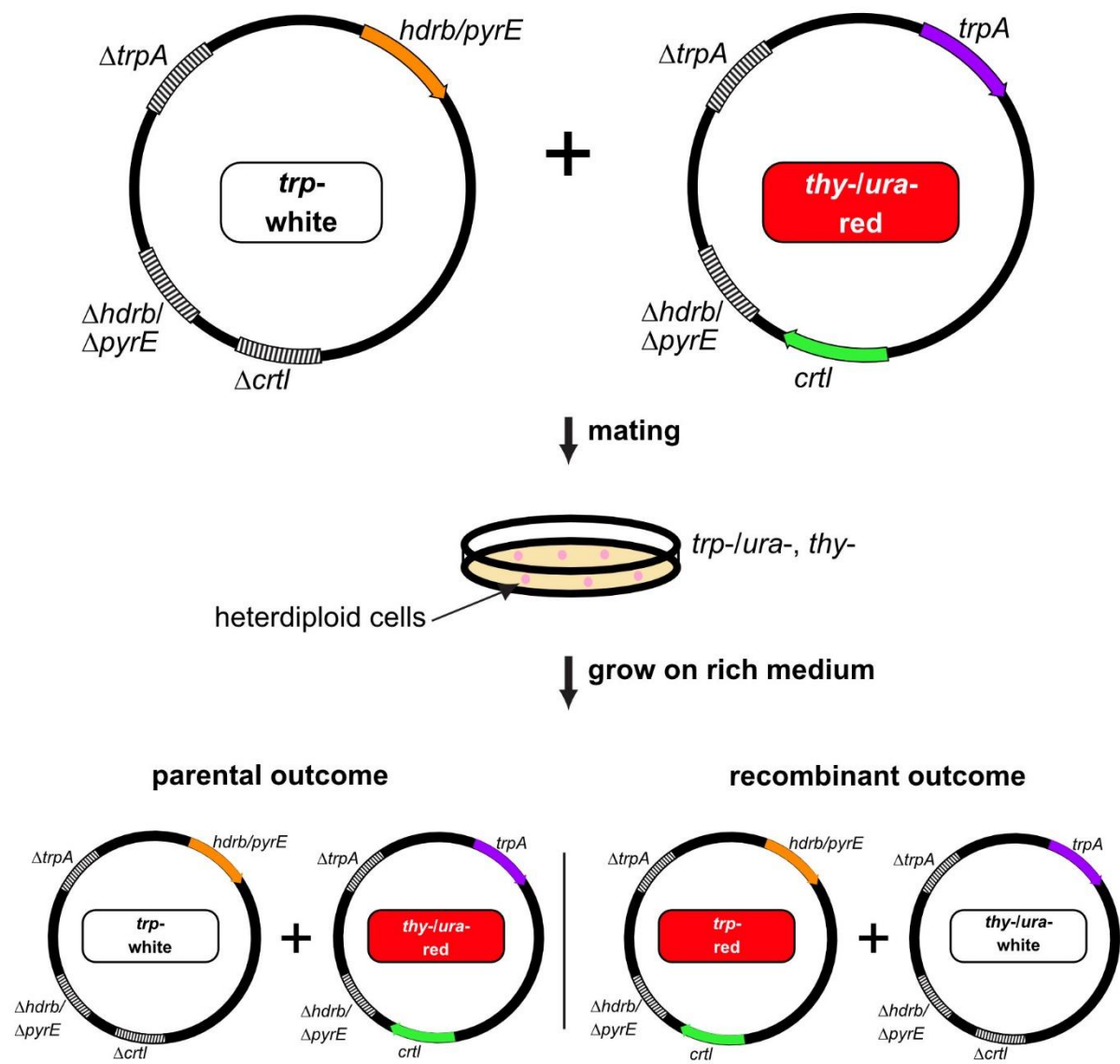

Supplement: jkac306_Supplementary_Data [file jkac306_supplementary_data.zip › Supplementary_Figure_1_G3-2022-403706.pdf]
